# Characterization, Applications and New Technologies of Civil Engineering Materials and Structures

**DOI:** 10.3390/ma17092058

**Published:** 2024-04-27

**Authors:** Wensheng Wang, Qinglin Guo, Jue Li

**Affiliations:** 1 College of Transportation, Jilin University, Changchun 130022, China; 2 School of Civil Engineering, Hebei University of Engineering, Handan 056038, China; 3 College of Traffic & Transportation, Chongqing Jiaotong University, Chongqing 400074, China

## 1. Introduction

With the continuous development of large-scale maintenance of infrastructure, accurate, reasonable, and efficient mechanical behavior evaluation and performance prediction of civil materials and structures have become the keys to improving service durability and intelligent maintenance management for infrastructure. The multi-component composition, multi-scale characteristics, and multi-field dependence of civil materials lead to extremely complex mechanical behaviors. The phenomenological method based on empirical tests is an important means to understand and evaluate civil materials, but its low efficiency and high consumption cannot meet the design and application requirements of civil materials. Numerical simulation has gradually become an important tool to study and understand the mechanical behavior of civil materials and structures, including the finite element method, discrete element method, molecular dynamics simulation, etc. In addition, the rapid development of numerical simulations has greatly promoted the modeling and simulation of civil materials. Artificial intelligence is known for including powerful computational techniques and is now being used more frequently by civil engineers to solve real problems related to civil materials and structures. This field is under rapid development, and numerous novel technologies have been proposed to characterize and evaluate the performance of civil materials and structures. Considering the above, the aim of this Special Issue is to bring together cutting-edge research and application. To share, present, and discuss innovative materials, structures, and characterization methods may help us further develop the technology used in civil engineering.

## 2. Highlights in the Present Issue

A total of twenty research papers and one systematic review are presented in this Special Issue, covering the application of solid wastes in civil engineering materials, environmental impact, material properties, mechanical behavior of civil engineering materials, etc. Multiple studies explored the use of industrial solid waste and various materials in enhancing soil, concrete, asphalt, and tunnel grouting materials, aiming to optimize their properties and processing parameters. Qiu et al. used an all-solid-waste binder composed of general industrial solid waste calcium carbide residue, ground granulated blast furnace slag, and fly ash instead of cement and combined with polypropylene fiber to strengthen the silty soil [1]. Lv et al. investigated the influence of recycled aggregate on different cement mortar pretreatment methods, including natural aggregate concrete and concrete with recycled aggregate after the wetting pretreatment and cement mortar pretreatment [2]. Pan et al. investigated the storage stability of crumb rubber asphalt with epoxidized soybean oil and polyester fiber based on the response surface methodology [3]. Liu et al. explored the influence of material design parameters on the physical and mechanical properties of recycled asphalt to determine the optimal processing parameters using response surface methodology [4]. Jiang et al. presented a comprehensive review of the recent advancements in tunnel grouting materials using industrial solid waste [5]. Xu et al. used controlled low-strength materials for backfilling narrow spaces to achieve efficient and high-quality backfilling effects for pipeline trenches [6]. Liu et al. investigated the effects of foam densities, foaming agent types, and the presence of slurry on the pore structure and mechanical properties of foamed lightweight soils to evaluate their stability [7]. Zhao et al. examined the foam stability of different foaming agent dilution ratios by analyzing foam density, foaming ratio, settlement distance, and bleeding volume, developing and evaluating the effectiveness of foamed lightweight soil grouting material for goaf treatment [8]. Chong et al. investigated the influence of dry–wet cycles and sulfate attack on the performance of magnesium potassium phosphate cement as well as the effect of waterglass [9]. Xu et al. evaluated the effect of polyphosphoric acid on the ultraviolet aging resistance of styrene butadiene rubber-modified asphalt [10]. Klimczak et al. used the respective methodology to identify the parameters of the well-established Bodner–Partom material model [11]. Tan et al. investigated the applicability and reliability of ultrasonic testing technology in evaluating the performance (ultrasonic, indirect tensile, compression, and dynamic modulus tests) of asphalt mixtures at various temperatures [12]. Diao et al. selected hydrated lime and basalt fiber to prepare a modified asphalt mixture to enhance the fatigue resistance of aging asphalt pavement [13]. Yang et al. conducted monotonic tensile tests on three kinds of stone-mastic asphalt mixtures to determine the optimum dosage of each kind of fiber [14]. Civil materials and structures are essential to engineering, but are very vulnerable to harsh environments, freeze–thaw actions, loading, etc. These factors can cause damage to civil structure and infrastructure by exerting negative influences on the mechanical and functional properties of civil materials (e.g., asphalt and cement concrete). Liu et al. discussed a series of tensile and puncture tests of geotextiles under different low temperatures and at different moisture content levels [15]. Haimei et al. clarified the influence of moisture and aging on the healing ability of asphalt–aggregate bonding interfaces based on laboratory experiments and the meso-finite element method [16]. Yang et al. evaluated the effect of the number of drains per pile and the orientation of the drains relative to the direction of shaking [17]. Tang et al. studied the flexural performance of the combined structure of steel pipe and steel slag powder in ultra-high-performance concrete via a pure bending test and a finite element simulation [18]. Wang et al. investigated the strength and failure characteristics of silty mudstone using different stress paths through triaxial unloading tests [19]. Obara et al. carried out a parametric analysis of tensegrity structures subjected to periodic loads, determining the main region of dynamic instability [20]. Zhang et al. investigated the temperature effect, including hydration heat and the sunshine temperature effect, of the construction process of a rigid frame-tied steel box arch bridge [21].
